# Association Between Sugar‐Sweetened Beverage Consumption and All‐Cause Mortality in Individuals With and Without Metabolic Syndrome: A Prospective Cohort Study

**DOI:** 10.1002/hsr2.73438

**Published:** 2026-10-26

**Authors:** Tingting Lv, Xintian Ren, Yanfang Tan, Min Li

**Affiliations:** ^1^ Liver Center, Beijing Friendship Hospital Capital Medical University Beijing China; ^2^ Clinical Epidemiology and EBM Unit, Beijing Friendship Hospital Capital Medical University; Beijing Clinical Research Institute Beijing China

**Keywords:** all‐cause mortality, metabolic syndrome, sugar‐sweetened beverages

## Abstract

**Background and Aims:**

To examine the association between sugar‐sweetened beverage (SSB) intake and all‐cause mortality among U.S. adults with and without metabolic syndrome (MetS), and to identify potential nonlinear dose‐response relationships and high‐risk subpopulations.

**Methods:**

We analyzed data from 26,885 U.S. adults aged 20–79 years who participated in NHANES 2003–2018. MetS was defined based on the NCEP ATP III criteria. SSB intake was assessed using a single 24‐h dietary recall and quantified as total beverage weight (g/day), energy intake (kcal/day), and sugar intake (g/day). Cox proportional hazards models and restricted cubic splines were used to assess the association of SSB intake and mortality in MetS and non‐MetS groups.

**Results:**

Among 8,656 participants with MetS and 18,229 without, total weight and energy intake from SSBs showed non‐linear association with the risk of all‐cause mortality in the MetS participants (*P*_non‐linear_ < 0.05), with inflection points of approximately 950 g/day and 90 kcal/day, respectively. Above this level, each standard deviation (SD) increment was associated with 14% (HR: 1.14, 95% CI: 1.01–1.29) and 18% (HR: 1.18, 95% CI: 1.08–1.30) higher mortality risk. For SSB‐derived sugar, the non‐linear trend missed statistical significance (*p* = 0.07), though risk nadir appeared at ~20 g/day, with subsequent SD increments linked to a 19% risk increase (HR: 1.19, 95% CI: 1.09–1.30). No linear or non‐linear association was presented among individuals without MetS.

**Conclusion:**

High SSB intake is associated with increased all‐cause mortality in adults with MetS, with nonlinear threshold effects.

## 1 Introduction

The global burden of metabolic syndrome (MetS), a cluster of metabolic abnormalities including central obesity, dyslipidemia, hypertension, and hyperglycemia, has been rising steadily in both developed and developing countries, now affecting approximately one in four adults worldwide [1, 2]. As a major risk factor for cardiovascular disease, type 2 diabetes, and premature mortality, MetS has emerged as a critical target for preventive interventions in dietary and public health research [3].

Among the modifiable lifestyle factors contributing to MetS, sugar‐sweetened beverages (SSBs) are of particular concern. As a leading global source of added sugars, excessive SSB consumption has been consistently linked to adverse metabolic outcomes, including obesity, insulin resistance, and type 2 diabetes [4, 5, 6]. Several large‐scale studies have identified a positive association between SSB intake and the development of MetS, suggesting that frequent consumption of sugary beverages may initiate or exacerbate metabolic dysregulation [3, 7, 8]. Because SSBs may affect multiple interrelated metabolic processes rather than a single marker alone, MetS provides a clinically meaningful phenotype for evaluating combined metabolic vulnerability associated with SSB exposure. Although SSB consumption has declined in the United States (U.S.), NHANES data from 2017 to 2018 showed that 65.3% of non‐Hispanic White adults and 77.8% of Hispanic adults consumed SSBs on a given day [9], and recent NHIS 2022 data reported past‐month SSB consumption in 82.69% of U.S. adults [10]. Notably, SSB intake was not merely occasional.

While the role of SSBs in the development of MetS is well documented, less is known about the relationship between SSB intake and mortality among individuals who already have MetS. Most prior research has focused on general populations or specific high‐risk subgroups, such as individuals with inflammatory bowel disease or chronic kidney disease [11, 12, 13]. However, evidence regarding mortality outcomes in those with established metabolic abnormalities is limited. For example, a recent U.S. study reported that high SSB intake was associated with a nearly three‐fold increase in cardiovascular mortality among adults with metabolic dysfunction‐associated steatotic liver disease, but no significant link was observed with all‐cause mortality [14]. Moreover, emerging evidence suggests that the association between SSB consumption and mortality may be nonlinear, characterized by threshold or inflection points beyond which health risks escalate markedly [5]. This possibility may be particularly relevant in individuals with MetS, a population in whom dietary modification is an important component of cardiometabolic risk management [15].

To address these gaps, we conducted a nationally representative prospective cohort study using data from the U.S. National Health and Nutrition Examination Survey (NHANES) to evaluate the associations between SSB consumption and all‐cause mortality among individuals with and without MetS. We further explored dose‐response relationships, potential nonlinear thresholds, and effect modification across key demographic and metabolic subgroups.

## 2 Methods

### 2.1 Study Design and Participants

The NHANES is an ongoing, nationally representative survey designed to monitor the health and nutritional status of the U.S. population [16]. It collects comprehensive information through demographic interviews, physical examinations, laboratory assessments, and dietary surveys, with data released biennially [17]. The NHANES study protocol was approved by the Research Ethics Review Board of the National Center for Health Statistics, and all participants provided written informed consent.

This study utilized data from NHANES cycles conducted between 2003 and 2018. We included participants aged 20 to 79 years who had complete 24‐h dietary recall data. As shown in Figure 1, among 44,790 individuals aged 20–79 years, 9715 participants without valid dietary recall data and 8190 participants with missing information required to define MetS status or without mortality follow‐up data were excluded. The final analytic sample included 26,885 participants, comprising 8656 individuals with MetS and 18,229 without MetS.

**Figure 1:**
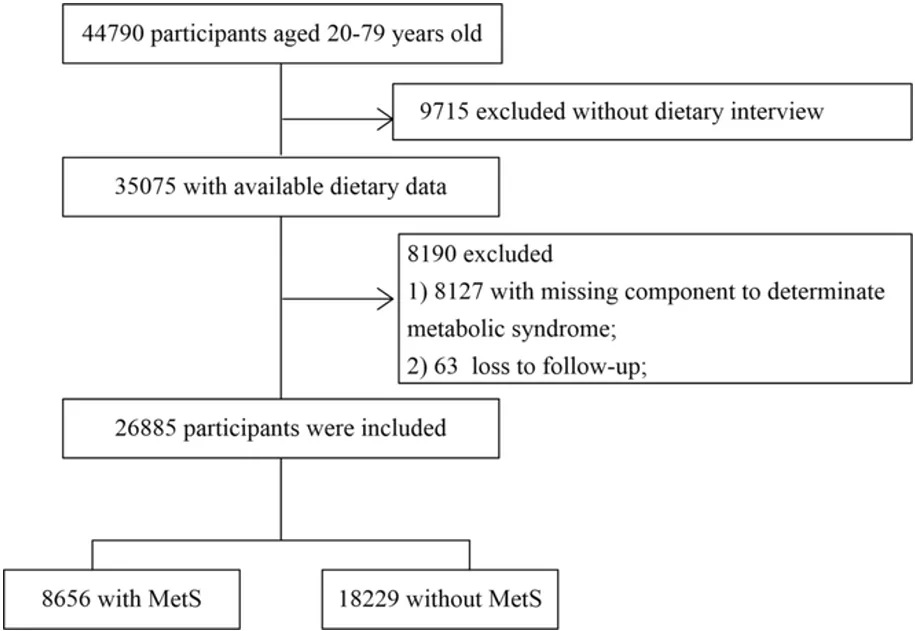
Flowchart of study participant selection from the NHANES cohort. MetS, metabolic syndrome. *Note:* MetS classification accounted for partial missing data to minimize selection bias: (1) MetS was confirmed if ≥ 3 components were present, regardless of missing items; (2) Non‐MetS was confirmed if the sum of existing positive components plus all missing items could not mathematically reach 3. Remaining indeterminate cases were excluded.

### 2.2 Definition of Metabolic Syndrome

MetS was defined according to the criteria of the National Cholesterol Education Program Adult Treatment Panel III (NCEP ATP III) as the presence of at least three of the following five components [18]: (1) waist circumference ≥ 88 cm in women and ≥ 102 cm in men (≥ 80 cm in women and ≥ 90 cm in men if Asian ancestry); (2) triglyceride level ≥ 150 mg/dL; (3) high‐density lipoprotein cholesterol (HDL‐C) < 40 mg/dL in men or < 50 mg/dL in women; (4) blood pressure ≥ 130/85 mmHg or use of antihypertensive medication; and (5) fasting glucose ≥ 100 mg/dL. Participants with partial missing data in MetS components were strictly evaluated; they were classified and retained only if their available data were mathematically sufficient to confirm or rule out the diagnosis, while those with indeterminate status were excluded.

### 2.3 Assessment of Sugar‐Sweetened Beverage Intake

Dietary intake was assessed using a 24‐h dietary recall interview. SSBs were defined according to standard NHANES protocols and included soft drinks, fruit drinks, nutritional drinks, sweetened teas and coffees, sports and energy drinks, smoothie, and carbonated or bottled water. Excluded beverages included 100% fruit juice, alcoholic beverages, fluid replacement, flavored milks, and self‐sweetened beverages [19]. Three key exposure variables were derived: (1) total sugar intake from SSBs (g/day); (2) total gram of SSBs consumed (g/day); (3) total energy intake from SSBs (kcal/day).

## 3 Mortality Outcomes and Covariates

The primary outcome was all‐cause mortality, determined through linkage to the National Death Index. Follow‐up time was calculated from the date of the NHANES interview to the date of death or December 31, 2019, whichever came first. Associations between SSB intake and all‐cause mortality were assessed separately among participants with and without MetS. Covariates included age, sex, race/ethnicity, educational attainment, marital status, household income, smoking status, alcohol consumption, body mass index (BMI), medical history (including congestive heart failure, coronary heart disease, stroke, hypertension, diabetes, and cancer), and NHANES cycle year.

## 4 Statistical Analysis

All analyses incorporated NHANES‐provided sample weights to account for the complex, multistage survey design. Continuous variables were expressed as medians and interquartile ranges and compared using the Wilcoxon rank‐sum test. Categorical variables were reported as weighted percentages with 95% confidence intervals (CIs) and compared using the Rao‐Scott adjusted chi‐square test to account for complex survey design.

Weighted Cox proportional hazards models were used to estimate hazard ratios (HRs) and 95% CIs for the association between each SSB intake indicators and all‐cause mortality in MetS and non‐MetS groups. Restricted cubic spline (RCS) was employed to assess potential nonlinear relationships between each SSB intake indicators and all‐cause mortality. In the case of a nonlinear relationship (P_non‐linear_ < 0.05), a threshold was identified at the point where the slope of the spline curve changed. Subsequently, a two‐piecewise Cox proportional hazards model was fitted to examine potential threshold effects. Stratified analyses were conducted to explore effect modification by age, sex, and individual MetS components. All multivariable models were adjusted for the aforementioned covariates.

Statistical analyses were performed using R (version 4.2.2; R Foundation for Statistical Computing, Vienna, Austria) and SAS version 9.4 (version 9.4; SAS Institute, Cary, NC, USA). A two‐sided *p* < 0.05 was considered statistically significant.

## 5 Results

### 5.1 Baseline Characteristics

Compared with participants without MetS, those with MetS were older and had a greater cardiometabolic burden, with higher median age (54.59 vs. 40.85 years), BMI (31.90 vs. 25.51 kg/m^2^), waist circumference (109.17 vs. 90.86 cm), fasting blood glucose (108.80 vs. 94.99 mg/dL), and HbA1c (5.66% vs. 5.27%). The prevalence of hypertension (84.55% vs. 30.09%) and diabetes (30.48% vs. 4.73%) was markedly higher in the MetS group. During a median follow‐up of 8.33 years, MetS participants also demonstrated a significantly elevated risk of all‐cause mortality (13.35% vs. 5.94%). In addition, they were more likely to have lower educational attainment and lower household income (Table 1).

**Table 1:** Characteristics of study participants by metabolic syndrome status.

Variables	MetS	No MetS	*p* value
Age, years	54.59 (43.45–65.38)	40.85 (29.14–54.42)	< 0.01
Male, %	4063 [48.42 (46.95, 49.89)]	9478 [50.44 (49.60, 51.29)]	0.02
Race, %			0.01
Mexican American	1514 [8.20 (6.74–9.67)]	2962 [8.53 (7.30–9.77)]	
Other Hispanic	816 [4.75 (3.81–5.68)]	1683 [5.54 (4.74–6.34)]	
Non‐Hispanic White	4087 [71.34 (68.68–73.99)]	8233 [68.82 (66.46–71.17)]	
Non‐Hispanic Black	1550 [9.39 (8.13–10.64)]	3634 [10.25 (9.06–11.44)]	
Other Race	689 [6.33 (5.43–7.22)]	1717 [6.86 (6.08–7.64)]	
Education, %			< 0.01
High school or less	4747 [46.67 (44.54–48.80)]	8246 [36.79 (35.01–38.58)]	
Some college or higher	3898 [53.33 (51.20–55.46)]	9971 [63.21 (61.42–64.99)]	
Family income, %			< 0.01
Low	2610 [21.34 (19.82–22.86)]	5005 [19.56 (18.29–20.83)]	
Medium	3167 [35.70 (34.12–37.29)]	31.69 [31.69 (30.29–33.10)]	
High	2150 [36.50 (34.31–38.69)]	5602 [42.40 (40.53–44.27)]	
Marital status, %			0.01
Married/Cohabiting	5381 [65.21 (63.63–66.78)]	11041 [62.65 (61.19–64.12)]	
Single/Divorced/Widowed	3273 [34.79 (33.22–36.37)]	7182 [37.35 (35.88–38.81)]	
BMI, kg/m^2^	31.90 (28.65–36.54)	25.51 (22.67–28.79)	< 0.01
WC, cm	109.17 (101.72–118.98)	90.86 (82.20–99.43)	< 0.01
Smoking, %	4395 [52.57 (51.03–54.12)]	8255 [45.97 (44.55–47.39)]	< 0.01
Alcohol consumer, %	5197 [67.36 (65.47–69.25)]	12661 [78.64 (77.36–79.92)]	< 0.01
SBP, mmHg	129.60 (118.20–139.91)	115.39 (107.51–123.90)	< 0.01
DBP, mmHg	73.27 (64.56–81.73)	69.27 (62.57–75.58)	< 0.01
FBG, mg/dL	108.80 (101.83–121.92)	94.99 (89.92–100.95)	< 0.01
HbA1c, %	5.66 (5.35–6.16)	5.27 (5.03–5.50)	< 0.01
Insulin, pmol/L	86.79 (57.53–133.28)	42.40 (28.22–65.22)	< 0.01
TC, mg/dL	193.21 (164.92–224.27)	190.63 (164.83–218.57)	< 0.01
TG, mg/dL	159.09 (107.90–216.05)	87.38 (63.10–118.19)	< 0.01
HDL‐C, mg/dL	40.85 (35.20–47.91)	55.57 (46.96–66.41)	< 0.01
LDL‐C, mg/dL	112.93 (89.28–138.62)	110.47 (89.20–134.30)	< 0.01
Hypertension, %	7374 [84.55 (83.29–85.80)]	6018 [30.09 (29.14–31.04)]	< 0.01
Diabetes, %			< 0.01
Normal	1451 [19.96 (18.56–21.36)]	12182 [72.81 (71.75–73.88)]	
Pre‐diabetes	4068 [49.55 (48.04–51.07)]	4735 [22.46 (21.48–23.44)]	
Diabetes	3134 [30.48 (29.08–31.89)]	1302 [4.73 (4.31–5.15)]	
Cancer, %	1106 [13.62 (12.47–14.77)]	1319 [7.47 (6.94–8.00)]	< 0.01
Congestive heart failure, %	509 [4.81 (4.20–5.42)]	299 [1.16 (0.98–1.34)]	< 0.01
Coronary heart disease, %	622 [6.69 (5.97–7.42)]	463 [1.98 (1.67–2.29)]	< 0.01
Stroke, %	533 [5.10 (4.49–5.72)]	412 [1.70 (1.48–1.91)]	< 0.01
All‐cause mortality, %	1434 [13.35 (12.38–14.32)]	1601 [5.94 (5.43–6.45)]	< 0.01

*Note:* Continuous variables are presented as weighted interquartile Range, Categorical variables were presented as unweighted number and weighted percentage with 95% confidence interval.

Abbreviations: BMI, body mass index; DBP, diastolic blood pressure; FBG, fasting blood glucose; HbA1c, hemoglobin A1c; HDL‐C, high‐density lipoprotein cholesterol; LDL‐C, low‐density lipoprotein cholesterol; MetS, metabolic syndrome; SBP, systolic blood pressure; TC, total cholesterol; TG, triglycerides; WC, waist circumference.

With respect to SSB intake, those with MetS consumed significantly greater total beverage weight (867.41 g vs. 790.40 g, *p* < 0.01). However, Median energy intake and sugar intake from SSBs was slightly lower in the MetS group (91.32 kcal vs. 117.83 kcal; 18.34 g vs. 22.84 g), but the difference was not statistically significant (*p* = 0.19; *p* = 0.80).

### 5.2 Association of SSB Intake With All‐Cause Mortality

RCS analyses revealed varying non‐linear patterns between specific domains of SSB consumption and all‐cause mortality within the MetS cohort (Figure 2). For total weight and energy from SSBs, distinct threshold effects were identified at approximately 950 g/day and 90 kcal/day, respectively (*P*_non‐linear_ < 0.05). Above this level, each standard deviation (SD) increment was associated with 14% and 18% increase in mortality risk, respectively (HR = 1.14, 95% CI: 1.01–1.29, *p* = 0.04; HR = 1.18, 95% CI: 1.08‐1.30, *p* < 0.01). A non‐significant, non‐linear trend was observed for sugar intake from SSBs (*p* = 0.07); visually, however, the risk reached its nadir at ~ 20 g/day, past each SD increment was associated with heightened mortality (HR = 1.19; 95% CI: 1.09–1.30) (Table 2).

**Figure 2:**
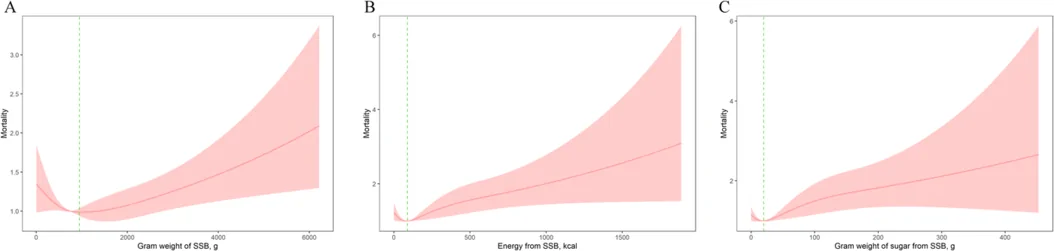
Non‐linear associations of SSB consumption with all‐cause mortality among participants with metabolic syndrome. Restricted cubic splines within weighted Cox proportional hazards regression models describe the multi‐variable adjusted Hazard Ratios (solid lines) and 95% confidence intervals (shaded areas) for (A) total gram weight of SSBs, (B) energy intake from SSBs, and (C) sugar from SSBs. The green dashed lines indicate the respective inflection points (thresholds). Models were adjusted for age, sex, race, education level, marital status, household income, smoking status, alcohol consumption, body mass index, medical history (congestive heart failure, coronary heart disease, stroke, hypertension, diabetes, and cancer), and NHANES cycle. NHANES, National Health and Nutrition Examination Survey; SSB, sugar‐sweetened beverage.

**Table 2:** Analysis of the threshold effect of sugar‐sweetened beverages and mortality risk among US Adults.

Per 1 SD	Gram weight of SSBs, g		Energy from SSBs, kcal		Sugar intake from SSBs, g	
	Adjusted HR (95% CI)	*p* value	Adjusted HR (95%CI)	*p* value	Adjusted HR (95%CI)	*p* value
MetS						
Fitting by the standard Cox model	1.05 (0.95–1.15)	0.38	1.14 (1.05–1.23)	< 0.01	1.13 (1.05–1.22)	< 0.01
*p* value for nonlinearity		< 0.01		0.03		0.07
Inflection point	950		90		20	
Intake ≤ Inflection point	0.94 (0.87–1.02)	0.14	0.95 (0.85–1.06)	0.35	0.97 (0.88–1.08)	0.60
Intake > Inflection point	1.14 (1.01–1.29)	0.04	1.18 (1.08–1.30)	< 0.01	1.19 (1.09–1.30)	< 0.01
No MetS						
Fitting by the standard Cox model	1.07 (0.99–1.16)	0.08	1.09 (0.99–1.21)	0.08	1.07 (0.97–1.17)	0.16
*p* value for nonlinearity		0.20		0.42		0.61

*Note:* HR was calculated after adjusting for age, sex, race, education level, marital status, household income, smoking status, alcohol, BMI, medical history (congestive heart failure, coronary heart disease, stroke, hypertension, diabetes, cancer), and NHANES cycle.

Abbreviations: CI, confidence interval; HR, hazard ratio; MetS, metabolic syndrome; NHANES, National Health and Nutrition Examination Survey; SD, standard deviation; SSBs, sugar‐sweetened beverages.

In contrast, standard Cox models for participants without MetS yielded a marginal upward trend for both SSB gram weight and energy intake, though neither reached statistical significance (Table 2). And no significant nonlinear associations were identified (Supporting Information S1: Figure 1).

### 5.3 Subgroup Analyses Among Mets Participants

Participants aged < 65 years old and males reported higher SSB intake (Supplementary Table 1). However, mortality risks were more pronounced among older adults (HR for energy = 1.15; sugar = 1.13) and females (HR for energy = 1.23; sugar = 1.23; all *p* < 0.05). Significant interactions were observed for sex (*p* = 0.02 for sugar), indicating a stronger relative effect of sugar from SSBs on mortality in females.

Across MetS components, individuals with metabolic abnormalities, such as abdominal obesity, low HDL, and hypertriglyceridemia consumed higher total beverage weight (*p* < 0.05), despite variations in sugar and energy intake. These groups consistently showed elevated mortality risks associated with SSB consumption: abdominal obesity (HR for energy = 1.11; sugar = 1.10), low HDL (HR for energy = 1.20; sugar=1.16), impaired glucose tolerance (HR for energy = 1.13; sugar = 1.13), all *p* < 0.05. Interestingly, participants without hypertension (HR for weight = 1.12; energy = 1.14; sugar = 1.13) or without impaired glucose (HR for energy = 1.10) also showed significant risk, suggesting that harmful effects of SSBs may manifest before clinical diagnosis. Significant interactions were observed in low HDL cholesterol subgroup analysis (P for interaction = 0.03) (Table 3).

**Table 3:** Subgroup analysis of the association between sugar‐sweetened beverages and mortality risk.

Per 1 SD	Gram weight of SSBs, g			Energy from SSBs, kcal			Sugar intake from SSBs, g		
	HR (95% CI)	*p* value	*p* interaction	HR (95% CI)	*p* value	*p* interaction	HR (95% CI)	*p* value	*p* interaction
Age			0.32			0.80			0.86
≥ 65 years	1.08 (0.99–1.19)	0.09		1.15 (1.04–1.28)	0.01		1.13 (1.01–1.27)	0.04	
< 65 years	1.04 (0.96–1.13)	0.36		1.06 (0.97–1.15)	0.21		1.04 (0.96–1.12)	0.33	
Sex			0.63			0.06			0.02
Male	1.04 (0.96–1.13)	0.35		1.06 (0.97–1.16)	0.20		1.04 (0.96–1.12)	0.38	
Female	1.09 (1.00–1.18)	0.05		1.23 (1.11–1.37)	< 0.01		1.23 (1.11–1.37)	< 0.01	
Abdominal obesity			0.63			0.80			0.63
Yes	1.07 (0.98–1.15)	0.13		1.11 (1.02–1.21)	0.01		1.10 (1.02–1.20)	0.02	
No	1.05 (0.95–1.15)	0.35		1.09 (0.97–1.22)	0.17		1.06 (0.95–1.18)	0.28	
Low HDL cholesterol			0.13			0.03			0.06
Yes	1.10 (1.00–1.21)	0.06		1.20 (1.10–1.30)	< 0.01		1.16 (1.07–1.25)	< 0.01	
No	1.04 (0.95–1.13)	0.39		1.04 (0.95–1.13)	0.37		1.03 (0.95–1.12)	0.43	
Hypertriglyceridemia			0.59			0.70			0.57
Yes	1.01 (0.88–1.14)	0.94		1.13 (0.99–1.28)	0.06		1.12 (0.99–1.26)	0.07	
No	1.07 (0.97–1.18)	0.16		1.11 (0.97–1.28)	0.13		1.09 (0.96–1.23)	0.19	
Hypertension			0.28			0.83			0.70
Yes	1.02 (0.95–1.10)	0.56		1.11 (1.02–1.20)	0.01		1.08 (1.01–1.16)	0.03	
No	1.12 (1.01–1.23)	0.03		1.14 (1.02–1.27)	0.02		1.13 (1.02–1.25)	0.02	
Impaired fasting glucose			0.15			0.99			0.67
Yes	1.02 (0.93–1.11)	0.72		1.13 (1.01–1.27)	0.04		1.13 (1.01–1.26)	0.03	
No	1.08 (0.99–1.17)	0.07		1.10 (1.01–1.20)	0.02		1.08 (0.99–1.17)	0.07	

*Note:* HR was calculated after adjusting for age, sex, race, education level, marital status, household income, smoking status, alcohol, BMI, medical history (congestive heart failure, coronary heart disease, stroke, hypertension, diabetes, cancer), and NHANES cycle.

Abbreviations: CI, confidence interval; HR, hazard ratio; NHANES, National Health and Nutrition Examination Survey; SD, standard deviation; SSBs, sugar‐sweetened beverages.

### 5.4 High SSB Intake and Mortality Risk in MetS Subgroups

Among individuals exceeding intake thresholds (weight > 950 g/day, enereg > 90 kcal/day, sugar > 20 g/day), mortality risks remained elevated across key subgroups. Older adults (HR for weight = 1.33; energy = 1.41; sugar = 1.45), females (HR for energy = 1.31; sugar = 1.30), and those with low HDL (HR for weight = 1.17; energy = 1.19; sugar = 1.20) or impaired fasting glucose (HR for energy = 1.26; sugar = 1.23) were particularly vulnerable. Additionally, those without abdominal obesity (HR for weight = 1.53; energy = 1.59; sugar = 1.87) or without hypertension still exhibited strong associations, reinforcing that metabolic damage from SSBs may occur before overt MetS is detected (Supporting Information S1: Table S2).

## 6 Discussion

In this nationally representative cohort study of over 26,000 U.S. adults, we found that high SSB intake was significantly associated with increased all‐cause mortality in individuals with MetS. Importantly, this association appeared nonlinear, with potential inflection points above which mortality risk increased. Specifically, in the MetS population, mortality risk increased when SSB intake exceeded approximately 950 g/day of beverage weight, 90 kcal/day of SSBs‐derived energy, or 20 g/day of SSBs‐derived sugar, suggesting a threshold‐dependent pattern of risk. However, the nonlinear association for SSB‐derived sugar intake was not statistically significant, and this potential inflection point should be interpreted as exploratory. We also observed that individuals with MetS consumed a greater total weight of SSBs than those without MetS, despite similar SSBs‐derived sugar and energy intake, which may indicate differences in beverage consumption patterns that are not fully captured by sugar or energy content alone.

From a clinical and public health perspective, our findings add to existing evidence by identifying adults with MetS as a metabolically vulnerable group in whom higher SSB intake may be particularly relevant to mortality risk. In routine care, clinicians may consider assessing SSB intake more explicitly among patients with MetS, especially older adults, females, and individuals with abdominal obesity, low HDL cholesterol, or impaired fasting glucose. This may help inform future risk communication and dietary counseling, although the potential inflection points should not be interpreted as established intake thresholds or used as definitive clinical cutoffs. For public health practitioners, these findings may support prioritizing SSBs‐reduction strategies in adults with combined metabolic risk. However, because this study is observational, the identified thresholds should be interpreted as risk markers requiring further validation rather than definitive causal cutoffs for guidelines development.

While previous studies have consistently demonstrated a positive linear association between SSB intake and adverse health outcomes including all‐cause mortality, liver steatosis, and fibrosis, few have investigated potential nonlinear dose‐response relationships [5, 20, 21]. For instance, NHANES‐based research by Leung et al. reported a dose‐dependent increase in liver stiffness and steatosis with higher SSB consumption, although the analysis was based on linear trend models [20]. Similarly, in two large U.S. prospective cohorts, Malik et al. reported a dose‐response association between SSB consumption and total mortality, with pooled HRs increasing from 1.06 (95% CI: 1.03– 1.09) for two to six servings per week to 1.21 (95% CI: 1.13–1.28) for two or more servings per day, compared with less than one serving per month [22]. These studies underscore the metabolic burden associated with high SSB intake but do not address potential intake thresholds or inflection points. In contrast, our study used RCS to examine nonlinear associations and identified potential inflection points among individuals with MetS. Mortality risk appeared to increase above these levels, although the apparent threshold for SSB‐derived sugar intake remains uncertain given the nonsignificant nonlinear association. Notably, our findings align with a recent NHANES‐based CKD cohort study that also identified a nonlinear association between SSB intake and mortality, further supporting the relevance of threshold‐based dietary guidance in metabolically vulnerable populations [13].

SSBs‐related mortality risk may differ by age and sex among adults with MetS. In our subgroup analyses, older adults and females showed stronger associations between SSB intake and all‐cause mortality, despite generally lower reported intake levels. This finding should be interpreted cautiously, but it is consistent with NHANES‐based studies linking SSB intake to mortality in metabolically vulnerable adults, including those with metabolic dysfunction‐associated steatotic liver disease and chronic kidney disease [13, 14]. The California Teachers Study also reported a positive association between SSB intake and mortality among females [23], while a dose‐response meta‐analysis suggested greater SSBs‐related mortality risk in individuals aged ≥ 60 years [21]. These differences may reflect variations in metabolic reserve, body composition, hormonal status, and cumulative cardiometabolic burden, although the underlying mechanisms require further study.

Although the association of SSB consumption and mortality risk did not reach strict statistical significance among participants without MetS, a distinct directional consistency toward a higher mortality risk was still present, indicating potential population‐wide harm but with heightened sensitivity among those with MetS [24]. Moreover, participants without hypertension or impaired fasting glucose also demonstrated elevated mortality risks at higher SSB intake levels. These findings suggest that the adverse effects of excessive SSB consumption may precede overt metabolic disease. Supporting this notion, prior research has found that high SSB intake contributes to subclinical inflammation, ectopic fat storage, and insulin resistance even in individuals considered metabolically healthy. Stanhope et al. demonstrated that consuming fructose‐sweetened beverages, rather than glucose, led to significant increases in hepatic de novo lipogenesis and visceral adiposity, key markers of metabolic dysfunction [25]. Additionally, dietary sugar intake has been linked to systemic inflammation through increased free fatty acid synthesis in the liver, leading to oxidative stress and inflammatory mediators. Collectively, these data underline the importance of early dietary interventions targeting SSB consumption across the population, not solely in clinically at‐risk individuals.

Strengths of this study include its large, representative sample; objective linkage to mortality records; and comprehensive adjustment for confounders. We also employed spline regression to capture nonlinear patterns, which are often overlooked in dietary studies. However, the study has several limitations. First, dietary intake was assessed using self‐reported 24‐h dietary recall data, which may be subject to recall bias and may not fully capture usual long‐term intake. Second, residual confounding may persist despite multivariable adjustment. Third, cause‐specific mortality was not assessed in this analysis. Lastly, dietary intake was assessed at a single time point, which limits causal inference and may not capture changes in SSB consumption over time.

## 7 Conclusion

Our findings demonstrate a nonlinear, threshold‐dependent relationship between higher SSB intake and increased all‐cause mortality in adults with MetS. Notably, this risk is particularly pronounced among females. The observed inflection points may provide useful reference points for risk communication and dietary counseling.

## Author Contributions

**Tingting Lv:** writing – original draft, conceptualization, methodology, data curation. **Xintian Ren:** conceptualization, software, data curation, methodology. **Yanfang Tan:** conceptualization, methodology. **Min Li:** conceptualization, writing – review and editing, funding acquisition, project administration, supervision, formal analysis, data curation, software, methodology.

## Ethics Statement

The NHANES study protocol was approved by the Research Ethics Review Board of the National Center for Health Statistics, and all participants provided written informed consent.

## Conflicts of Interest

The authors declare no conflicts of interest.

## Transparency Statement

The corresponding author Min Li affirms that this manuscript is an honest, accurate, and transparent account of the study being reported; that no important aspects of the study have been omitted; and that any discrepancies from the study as planned (and, if relevant, registered) have been explained.

## Supporting information


Supporting File

## Data Availability

The data supporting the findings of this study are openly available in NHANES. These data were derived from the following resources available in the public domain: https://wwwn.cdc.gov/nchs/nhanes/Default.aspx.
