# Supplementary material for: Association Between Sugar‐Sweetened Beverage Consumption and All‐Cause Mortality in Individuals With and Without Metabolic Syndrome: A Prospective Cohort Study
Source: Health Sci Rep. 2026 Oct 26;9(11):e73438. doi: 10.1002/hsr2.73438 (PMC13698666; doi:10.1002/hsr2.73438)
Supplement: Supplementary file 1 [file HSR2-9-e73438-s001.docx]

**Association Between Sugar-Sweetened Beverage Consumption and All-Cause Mortality in Individuals With and Without Metabolic Syndrome: A Prospective Cohort Study**

Tingting Lv ^1^, Xintian Ren ^2^, Yanfang Tan ^2^, Min Li ^2^*

**Affiliations:**

^1^ Liver Center, Beijing Friendship Hospital, Capital Medical University, Beijing, China;

^2^ Clinical Epidemiology and EBM Unit, Beijing Friendship Hospital, Capital Medical University; Beijing Clinical Research Institute, Beijing, China;

**Contents:**

Supplementary Table 1. Intake level of sugar-sweetened beverages across subpopulations

Supplementary Table 2. Subgroup analysis of the association between sugar-sweetened beverages and mortality risk for MetS participants with sugar intake above the inflection point

Supplementary Figure 1. Weighted Cox proportional hazards regression model of sugar-sweetened beverages and mortality risk for participants without metabolic syndrome

Supplementary Table 1. Intake level of sugar-sweetened beverages across subpopulations

| Subgroup | Gram weight of SSBs, g | Energy from SSBs, kcal | Sugar intake from SSBs,g |
| --- | --- | --- | --- |
| Age |  |  |  |
| ≥ 65 years | 707.78 (371.90-1065.53)* | 14.43 (4.64-125.02)* | 0 (0-26.22)* |
| <65 years | 858.29 (496.00-1420.66) | 136.71 (13.44-302.82) | 28.58 (0-66.10) |
| Sex |  |  |  |
| Male | 953.57 (536.44-1509.97)* | 145.63 (13.49-324.42)* | 32.61 (0-72.26)* |
| Female | 719.79 (402.46-1149.06) | 76.87 (7.63-220.02) | 13.76 (0-46.87) |
| Abdominal obesity |  |  |  |
| Yes | 843.50 (495.07-1391.17)* | 92.20 (9.37-257.63)* | 16.73 (0-55.55)* |
| No | 785.98 (473.54-1321.97) | 131.57 (10.69-289.87) | 25.98 (0-64.50) |
| Low HDL cholesterol |  |  |  |
| Yes | 884.45 (511.59-1459.22)* | 143.85 (13.79-319.01)* | 31.68 (0-73.12)* |
| No | 782.71 (473.57-1305.06) | 98.87 (8.94-255.66) | 17.98 (0-54.80) |
| Hypertriglyceridemia |  |  |  |
| Yes | 880.25 (514.65-1452.89)* | 126.90 (10.45-282.22) * | 26.38 (0-65.49)* |
| No | 793.33 (473.59-1305.17) | 103.82 (9.22-270.08) | 19.27 (0-58.42) |
| Hypertension |  |  |  |
| Yes | 822.32 (495.79-1340.43) | 70.89 (7.58-242.42)* | 12.79 (0-52.08)* |
| No | 805.99 (479.81-1358.32) | 130.46 (12.34-288.40) | 25.79 (0-63.64) |
| Impaired fasting glucose |  |  |  |
| Yes | 824.74 (496.98-1361.36) | 84.76 (8.04-257.34)* | 15.75 (0-55.08)* |
| No | 806.92 (479.83-1347.50) | 119.63 (10.86-277.25) | 23.67 (0-61.69) |

*P<0.05. Abbreviation: SSB, sugar-sweetened beverages; HDL, high-density lipoprotein cholesterol.

Supplementary Table 2. Subgroup analysis of the association between sugar-sweetened beverages and mortality risk for MetS participants with sugar intake above the inflection point

| Per 1 SD | Gram weight of SSBs >950g | | |  | Energy from SSBs >90kcal | | |  | Sugar intake from SSBs >20g | | |
| --- | --- | --- | --- | --- | --- | --- | --- | --- | --- | --- | --- |
|  | HR(95%CI) | P value | P interaction |  | HR(95%CI) | P value | P interaction |  | HR(95%CI)* | P value | P interaction |
| Age |  |  | 0.22 |  |  |  | 0.06 |  |  |  | 0.05 |
| ≥ 65 years | 1.33 (1.08-1.63) | 0.01 |  |  | 1.41 (1.20-1.65) | <0.01 |  |  | 1.45 (1.17-1.79) | <0.01 |  |
| <65 years | 1.06 (0.88-1.28) | 0.52 |  |  | 1.13 (0.98-1.31) | 0.10 |  |  | 1.16 (1.03-1.30) | 0.02 |  |
| Sex |  |  | 0.14 |  |  |  | 0.06 |  |  |  | 0.05 |
| Male | 1.09 (0.93-1.26) | 0.28 |  |  | 1.14 (1.00-1.30) | 0.05 |  |  | 1.16 (1.04-1.28) | 0.01 |  |
| Female | 1.31 (0.99-1.73) | 0.06 |  |  | 1.31 (1.08-1.60) | 0.01 |  |  | 1.30 (1.08-1.56) | 0.01 |  |
| Abdominal obesity |  |  | 0.69 |  |  |  | 0.36 |  |  |  | 0.22 |
| Yes | 1.13 (0.99-1.28) | 0.07 |  |  | 1.13 (1.00-1.29) | 0.05 |  |  | 1.14 (1.02-1.27) | 0.02 |  |
| No | 1.53 (1.24-1.90) | <0.01 |  |  | 1.59 (1.33-1.89) | <0.01 |  |  | 1.87 (1.54-2.26) | <0.01 |  |
| Low HDL cholesterol |  |  | 0.37 |  |  |  | 0.66 |  |  |  | 0.79 |
| Yes | 1.17 (1.03-1.32) | 0.01 |  |  | 1.19 (1.07-1.32) | <0.01 |  |  | 1.20 (1.08-1.32) | <0.01 |  |
| No | 1.08 (0.81-1.42) | 0.60 |  |  | 1.13 (0.88-1.44) | 0.34 |  |  | 1.10 (0.85-1.43) | 0.45 |  |
| Hypertriglyceridemia |  |  | 0.87 |  |  |  | 0.23 |  |  |  | 0.23 |
| Yes | 1.17 (0.96-1.42) | 0.11 |  |  | 1.24 (1.04-1.47) | 0.02 |  |  | 1.21 (1.01-1.45) | 0.04 |  |
| No | 1.15 (0.84-1.56) | 0.38 |  |  | 1.29 (1.07-1.55) | 0.01 |  |  | 1.27 (0.99-1.63) | 0.06 |  |
| Hypertension |  |  | 0.28 |  |  |  | 0.55 |  |  |  | 0.59 |
| Yes | 1.10 (0.97-1.24) | 0.12 |  |  | 1.15 (1.04-1.28) | 0.01 |  |  | 1.15 (1.05-1.26) | <0.01 |  |
| No | 1.37 (0.99-1.89) | 0.06 |  |  | 1.58 (1.06-2.36) | 0.03 |  |  | 1.50 (1.00-2.24) | 0.05 |  |
| Impaired fasting glucose |  |  | 0.77 |  |  |  | 0.20 |  |  |  | 0.21 |
| Yes | 1.13 (0.96-1.32) | 0.14 |  |  | 1.26 (1.11-1.43) | <0.01 |  |  | 1.23 (1.07-1.43) | <0.01 |  |
| No | 1.15 (0.97-1.37) | 0.11 |  |  | 1.10 (0.92-1.32) | 0.30 |  |  | 1.13 (0.98-1.29) | 0.09 |  |

*HR was calculated after adjusting for age, sex, race, education level, marital status, household income, smoking status, alcohol, BMI, medical history (congestive heart failure, coronary heart disease, stroke, hypertension, diabetes, cancer), and NHANES cycle.

Abbreviation: MetS, Metabolic Syndrome; SD, standard deviation; SSB, sugar-sweetened beverages; HR, hazard ratio; CI, confidence interval; HDL, high-density lipoprotein cholesterol.

Supplementary Figure 1. Weighted Cox proportional hazards regression model of sugar-sweetened beverages and mortality risk for participants without metabolic syndrome

This model was performed after adjusting for age, sex, race, education level, marital status, household income, smoking status, alcohol, body mass index, medical history (congestive heart failure, coronary heart disease, stroke, hypertension, diabetes, cancer), and NHANES cycle. NHANES, National Health and Nutrition Examination Survey.
